# SUDOSCAN: A Simple, Rapid, and Objective Method with Potential for Screening for Diabetic Peripheral Neuropathy

**DOI:** 10.1371/journal.pone.0138224

**Published:** 2015-10-12

**Authors:** Dinesh Selvarajah, Tom Cash, Jennifer Davies, Adithya Sankar, Ganesh Rao, Marni Grieg, Shillo Pallai, Rajiv Gandhi, Iain D. Wilkinson, Solomon Tesfaye

**Affiliations:** 1 Department of Human Metabolism, University of Sheffield, Sheffield, United Kingdom; 2 Diabetes Research Unit, Sheffield Teaching Hospitals, Sheffield, United Kingdom; 3 Department of Neurophysiology, Sheffield Teaching Hospitals, Sheffield, United Kingdom; 4 Academic Unit of Radiology, University of Sheffield, Sheffield, United Kingdom; Sapienza, University of Rome, School of Medicine and Psycology, ITALY

## Abstract

Clinical methods of detecting diabetic peripheral neuropathy (DPN) are not objective and reproducible. We therefore evaluated if SUDOSCAN, a new method developed to provide a quick, non-invasive and quantitative assessment of sudomotor function can reliably screen for DPN. 70 subjects (45 with type 1 diabetes and 25 healthy volunteers [HV]) underwent detailed assessments including clinical, neurophysiological and 5 standard cardiovascular reflex tests (CARTs). Using the American Academy of Neurology criteria subjects were classified into DPN and No-DPN groups. Based on CARTs subjects were also divided into CAN, subclinical-CAN and no-CAN. Sudomotor function was assessed with measurement of hand and foot Electrochemical Skin Conductance (ESC) and calculation of the CAN risk score. Foot ESC (μS) was significantly lower in subjects with DPN [n = 24; 53.5(25.1)] compared to the No-DPN [77.0(7.9)] and HV [77.1(14.3)] groups (ANCOVA p<0.001). Sensitivity and specificity of foot ESC for classifying DPN were 87.5% and 76.2%, respectively. The area under the ROC curve (AUC) was 0.85. Subjects with CAN had significantly lower foot [55.0(28.2)] and hand [53.5(19.6)] ESC compared to No-CAN [foot ESC, 72.1(12.2); hand ESC 64.9(14.4)] and HV groups (ANCOVA p<0.001 and 0.001, respectively). ROC analysis of CAN risk score to correctly classify CAN revealed a sensitivity of 65.0% and specificity of 80.0%. AUC was 0.75. Both foot and hand ESC demonstrated strong correlation with individual parameters and composite scores of nerve conduction and CAN. SUDOSCAN, a non-invasive and quick test, could be used as an objective screening test for DPN in busy diabetic clinics, insuring adherence to current recommendation of annual assessments for all diabetic patients that remains unfulfilled.

## Introduction

The Toronto Consensus meeting defined Diabetic peripheral neuropathy (DPN) as a symmetrical and length-dependent sensorimotor polyneuropathy attributable to metabolic and microvessel alterations as a result of chronic hyperglycemia exposure [1]. It is now well recognised that DPN has major impact on quality of life, morbidity [2], mortality [3,4] and considerable health care costs [5]. Unfortunately, current bedside assessments for neuropathy such as the 10 gram monofilament [6], the Ipswich Touch Test [7], Vibratip [8] etc. are primarily aimed at screening for those at risk of foot ulceration and tend to diagnose DPN when it is well established. Late diagnosis hampers the benefits of early identification, the focus on early, intensified diabetes control, and the prevention of neuropathy-related sequelae [9]. A recent study has also found that there is significant variation on how clinical assessments are performed in practice and that the diagnosis of DPN is not always reproducible even when performed by experts [10]. Moreover, these clinical methods rely on the cognitive function of the subject and are not objective. This highlights the urgent need for an objective, quantitative screening test for DPN in clinical practice that overcomes the limitations of current methods.

Changes in peripheral autonomic nervous system function are an early manifestation of distal small fiber neuropathy [11]. Sudomotor dysfunction is one of the earliest detectable abnormalities in distal small fiber neuropathies [11]. Sweat glands are innervated by sudomotor, postganglionic, thin, unmyelinated cholinergic sympathetic C-fibres and a number of skin biopsy studies have shown a reduction in the epidermal C-nerve fibers in patients with diabetes [12]. Distal loss of sweating detected by the thermoregulatory sweat test correlated with subnormal quantitative sudomotor axon reflex response, an indication of a distal axonal neuropathy [13]. Therefore, assessment of sudomotor function may provide an attractive tool to evaluate peripheral small fibre neuropathy in diabetes [14].

**Fig 1:**
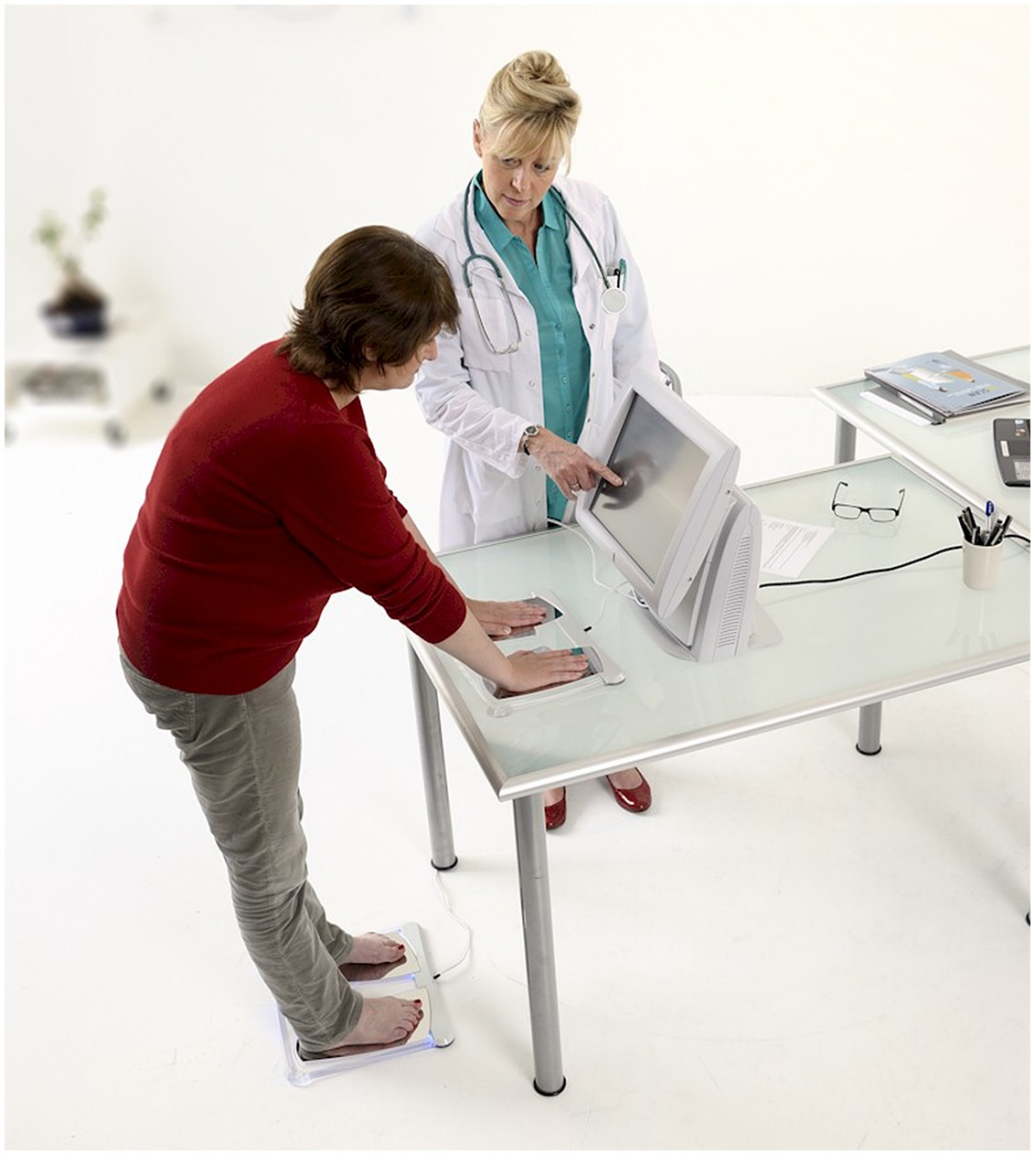
SUDOSCAN.

SUDOSCAN is a new device developed to provide a quick, non-invasive and reproducible, quantitative assessment of sudomotor function [Fig 1; 14–17]. Measurement is based on an electrochemical reaction between electrodes and chloride ions, after stimulation of sweat glands by a low-voltage current (<4volts) [14–17]. A measurement of conductance for the hands and feet, that are rich in sweat glands, is generated from the derivative current associated with the applied voltage [14]. SUDOSCAN shows good reproducibility in various physiological conditions. Furthermore, due to its focus on chloride concentrations it is less dependent on sweat rates than current methods used for assessment of sweat function [14–15]. However, none of the previous studies used gold standard electrophysiological assessment to define DPN. Hence, the aim of this study performed in subjects with type 1 diabetes (T1DM) was to evaluate if SUDOSCAN can reliably screen for DPN that was carefully characterized by using nerve conduction studies according to American Academy of Neurology guidelines.

## Materials and Methods

A total of 70 subjects (45 with T1DM and 25 healthy volunteers—HV) underwent detailed assessments including clinical and neurophysiological assessments to detect the presence and quantify the severity of DPN. DPN cases were defined according to established American Academy of Neurology consensus criteria using nerve conduction studies and clinical examination [18]. Cases with DPN had at least one neuropathic symptom or sign and at least one abnormal nerve conduction parameter in both sensory (sural) and motor (peroneal or tibial) nerves. Neuropathic symptoms were documented by completion of the NTSS-6 questionnaire which included numbness, burning, prickling paraesthesias, dysesthesias and allodynia [19]. Abnormal neurological signs included abnormal temperature, light touch, 10g monofilament (performed according to previously published criteria [20]) and absent or reduced knee/ankle reflexes. Neurological examination was performed according to the structured, validated Neuropathic Impairment Score of the Lower Limbs (NIS[LL]) questionnaire [21]. All subjects also underwent quantitative sensory assessments using the Computer Assisted Sensory Evaluation IV system (CASE IV, W.R. Electronics, Stillwater, MN). Vibration and cooling detection thresholds were acquired from the dorsal aspect of the right foot by employing standard techniques [22, 23]. Nerve conduction studies were performed at a stable skin temperature of 31°C and a room temperature of 24°C using a Medelec electrophysiological system (Synergy Oxford Instruments, Oxford, U.K.). The following nerve attributes were measured: a) sural sensory nerve action potential and conduction velocities, b) common peroneal nerve compound muscle action potential, conduction velocity and distal latency and c) tibial motor nerve distal latency. An overall neuropathy composite score (NCS—with a higher score indicating a more severe neuropathy) derived from transformed percentile points of abnormalities in sural nerve amplitude, tibial motor nerve distal latency, and peroneal motor nerve amplitude, latency and velocity, was calculated.

In addition all subjects also underwent 5 cardiovascular autonomic reflex tests (CART) according to the O’Brien protocol [24, 25]. Resting supine heart-rate and heart-rate responses to provocation tests (deep breathing, Valsalva and lying/standing), in addition to lying-to-standing blood pressure difference, were measured. Age adjusted normative data was used and a diagnosis of CAN was made if at least one out of the five tests were abnormal. An overall autonomic function test score (AFT score) was calculated based on age adjusted percentile abnormalities of CARTs. The higher the score the more severe is the CAN.

Finally, assessment of sudomotor function was performed using the SUDOSCAN test. This new and quick method is based on an electrochemical reaction between sweat chloride and stainless steel electrodes and has been validated in vitro and in previous clinical studies with assessment of reproducibility [14–17]. Patients placed the palms of their hands and the soles of their feet on stainless steel electrodes and an incremental low direct voltage (<4V) was applied for about 2 minutes. Electrochemical skin conductance (ESC), a measure of sudomotor function, is obtained from the ratio between the current that is measured and voltage applied. Quantitative results were expressed as ESC (microsiemens, μS) for the hands and feet, and a CAN risk score (CAN-RS) derived from the ESC values and demographic data (BMI and age) was calculated using an algorithm previously described [26]. Subjects with peripheral vascular disease were not excluded from the study. All subjects gave written informed consent for participating in this study which had prior ethics approval by the South Yorkshire and Humber Regional Ethics Committee.

### Statistics

Group demographic characteristics were compared using an analysis of variance (ANOVA). Subjects with T1DM were divided into two DPN groups according to criteria defined above. We used a univariate test (ANCOVA) to compare differences between groups (HV, No-DPN and DPN) by calculating mean foot and hand ESC per group adjusted for age and weight as fixed factors. A full factorial model was used with group difference as a contrast. T1DM subjects were then divided into two CAN groups (No-CAN and CAN) according to criteria defined above. A univariate test (ANCOVA) was used to compare differences between groups (HV, No-CAN and CAN) by calculating mean foot and hand ESC per group adjusted for age and weight as fixed factors. A full factorial model was used with group difference as a contrast.

The relation between mean foot and hand ESC and individual attributes of nerve function NCS and CART (AFT score) was examined in more detail among subjects with diabetes using Spearman’s Rank correlation coefficients. We also calculated the sensitivity, specificity and area under the ROC curve to examine the performance of SUDOSCAN measures to correctly identify subjects with DPN and CAN. Statistical analysis was done using statistical package SPSS 20.0.

## Results

Table 1 shows demographic and results of the neurophysiological and SUDOSCAN assessments. Subjects with DPN [52.1(9.7) years] were significantly older than the No-DPN [40.6(9.8), t-test p<0.001] and an older group of HV was recruited [48.1(16.4), ANOVA p = 0.01]. Subjects with diabetes [No-DPN 78.8(15.2) and DPN 83.3(14.4)] weighed more compared to HV [73.3(13.6), ANOVA p = 0.06]. As differences were observed in age and weight, analysis was adjusted based on these parameters.

**Table 1:** Demographics characteristics and SUDOSCAN outcomes of study subjects.

	**Healthy Volunteers**	**No DPN**	**DPN**	**p value**
N	25	21	24	
Sex (Male, %)	14 (56.0%)	12 (57.1%)	16 (66.6%)	0.71
Age (years)	48.1 (16.4)	40.6 (9.8)	52.1 (9.7)	0.01
Height (m)	1.71 (8.3)	1.71 (8.8)	1.72 (8.7)	0.84
Weight (Kg)	73.3 (13.6)	78.8 (15.2)	83.3 (14.4)	0.06
Systolic BP (mmHg)	137.3 (16.1)	130.8 (14.9)	134.2 (15.8)	0.38
HbA1c (mmol/mol)		73.3 (9.0)	77.8 (21.7)	0.38
Duration of Diabetes (years)		17.2 (9.9)	29.7 (13.4)	0.02
Vibration JND		15.7 (3.3)	20.0 (4.1)	0.001
Sural Amplitude (mV)		9.10 (3.1–27.5)	0.0 (0.0–7.0)	<0.001
Peroneal Velocity (m/s)		42.5 (36–52)	32.0 (0.0–44.2)	<0.001
Peroneal Amplitude (mV)		1.8 (0.20–6.2)	0.30 (0.0–4.0)	<0.001
Peroneal Distal Latency (mS)		4.80 (3.5–6.1)	4.75 (0.0–7.7)	0.41
Tibial Distal Latency (mS)		4.92 (3.4–6.4)	5.85 (0.0–8.1)	0.41
NCS score		1.00 (0.0–4.0)	10.5 (5.0–15.0)	<0.001
FESC (μS)	77.1 (14.3)	77.0 (7.9)	53.5 (25.1)	<0.001
HESC (μS)	64.4 (14.3)	66.4 (11.5)	49.2 (20.4)	0.001
CAN-RS (%)	21.1 (16.1)	17.6 (11.4)	32.1 (8.8)	0.001
	**Healthy Volunteers**	**No-CAN**	**CAN**	**p value**
n	25	25	20	
Sex (Male, %)	14 (56.0%)	13 (52.0%)	13 (65.0%)	0.26
Age (years)	48.1 (16.4)	43.6 (10.9)	50.6 (10.7)	0.20
Height (m)	1.71 (8.3)	1.71 (9.8)	1.71 (7.5)	0.98
Weight (Kg)	73.3 (13.6)	79.0 (15.5)	84.0 (13.8)	0.05
Systolic BP (mmHg)	137.3 (16.1)	132.0 (14.1)	133.4 (16.9)	0.47
HbA1c (mmol/mol)		73.8 (9.6)	78.1 (23.3)	0.41
Duration of Diabetes (years)		19.4 (11.6)	28.9 (13.7)	0.02
R-R Deep Breathing	1.29 (0.1)	1.32 (0.2)	1.14 (0.1)	<0.001
AFT Score	0.0	0.0	4.0 (1.0–12.0)	<0.001
FESC	77.1 (8.6)	72.1 (12.2)	55.0 (28.2)	<0.001
HESC	64.4 (14.3)	64.9 (14.4)	53.5 (19.6)	0.001
CAN-RS	21.1 (16.1)	20.8 (11.5)	31.0 (11.2)	0.02

JND, Just Noticeable Difference; NCS, Nerve Conduction Study; AFT, Autonomic Function Tests; FESC, Foot Electrochemical Skin Conductance; HESC, Hand Electrochemical Skin Conductance; CAN, Cardiac Autonomic Neuropathy, CAN-RS, Cardiac Autonomic Neuropathy Risk Score.

**Fig 2:**
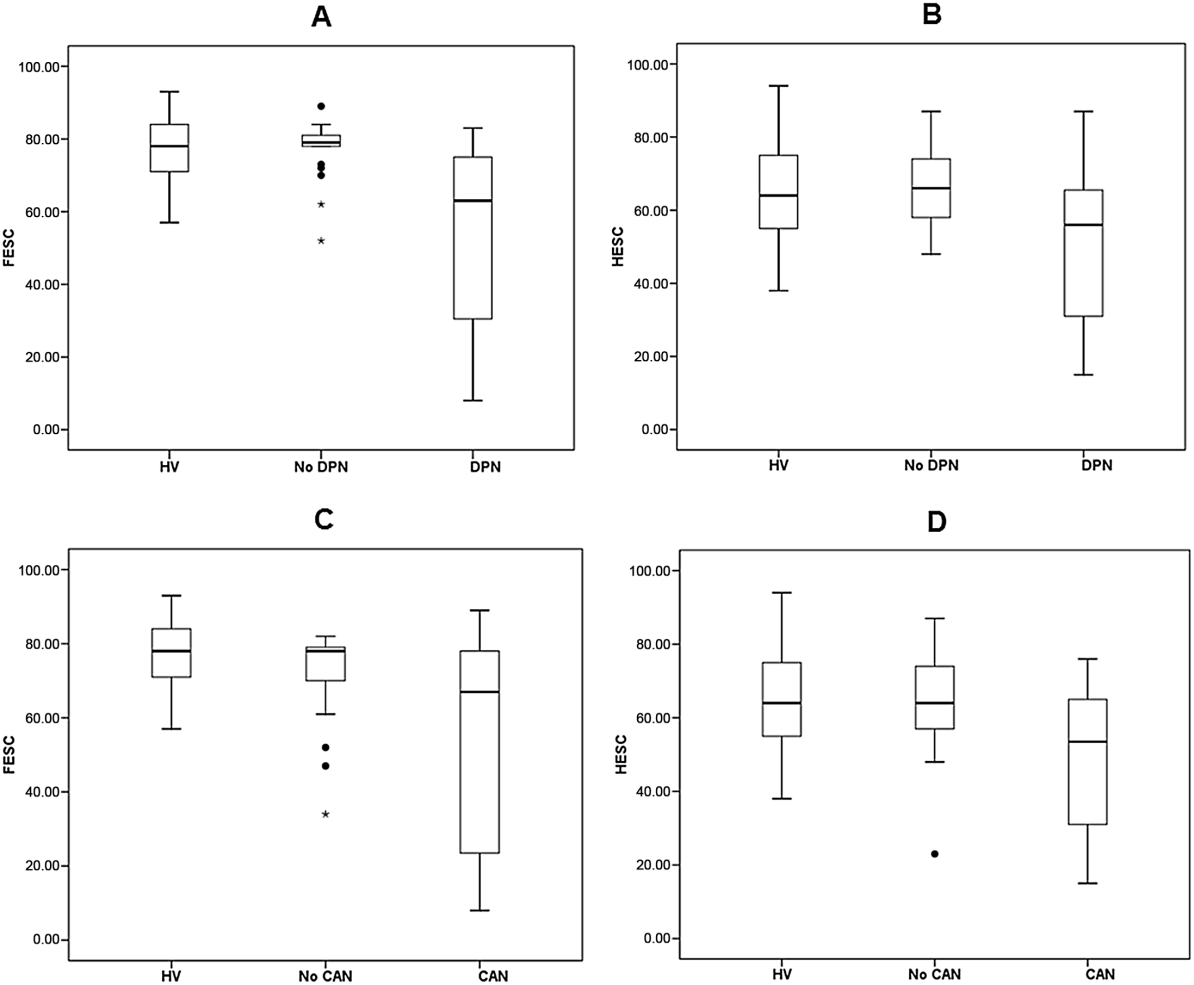
Box and whisker plots of SUDOSCAN outcomes of each study group. A, Foot Electrochemical Skin Conductance (FESC, μS) in healthy volunteers and subjects with type 1 diabetes divided into subjects with no diabetic peripheral neuropathy (No DPN) and DPN; B, Hand Electrochemical Skin Conductance (HESC, μS) in HV, No DPN and DPN groups; C, FESC in HV and subjects with type 1 diabetes divided into subjects with no Cardiac Autonomic Neuropathy (No CAN) and CAN; D, HESC in HV, No CAN and CAN groups.

**Fig 3:**
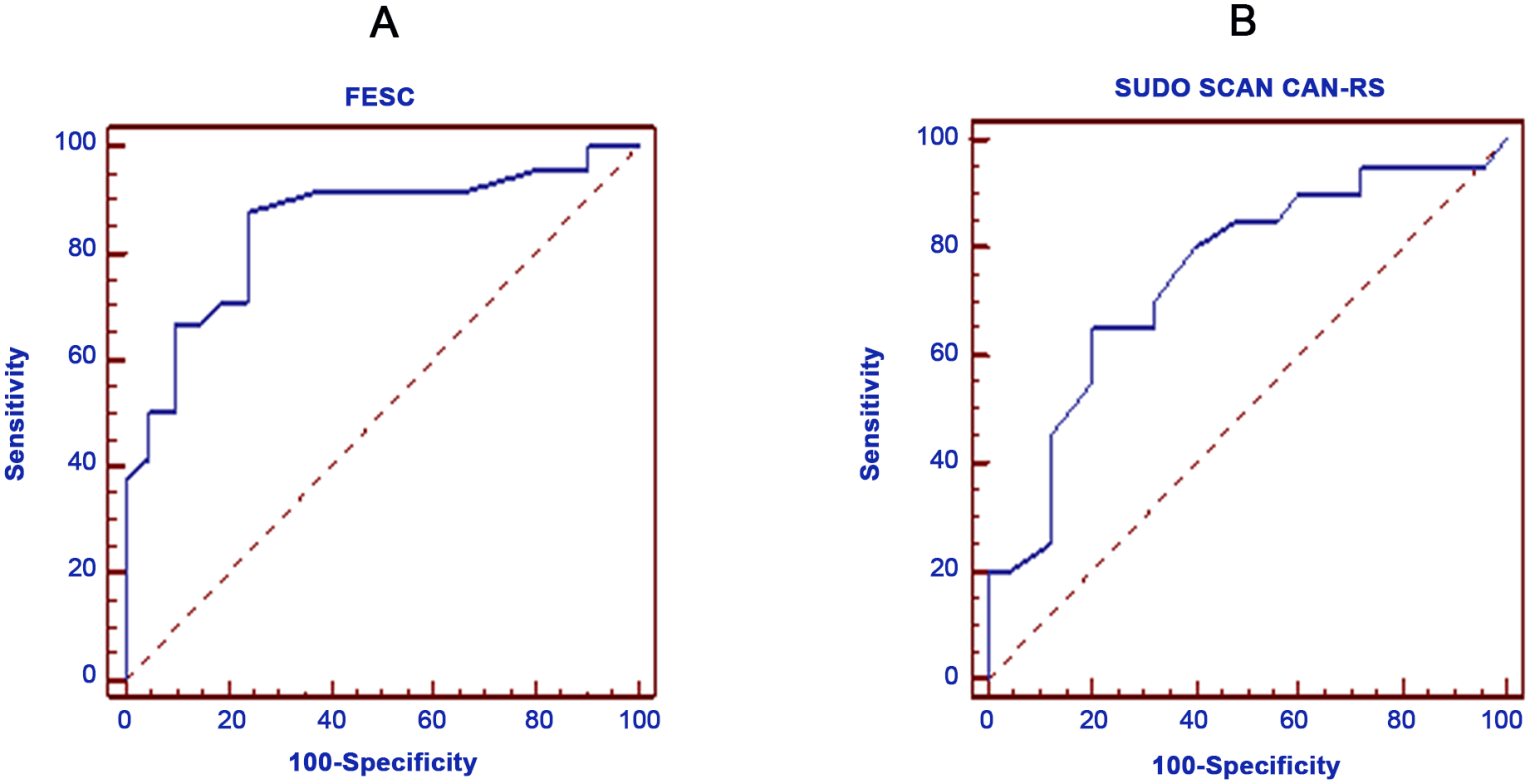
Graphic presentation of the diagnostic performance of (A) Foot Electrochemical Skin Conductance (FESC, μS) and (B) Cardiac Autonomic Neuropathy Risk Score (CAN-RS) by Receiver Operating Curve (ROC) analysis for diabetic peripheral neuropathy (A) and cardiac autonomic neuropathy (B, CAN).

Both foot and hand ESC demonstrated strong correlation with individual parameters and composite scores of CAN and NCS scores (Table 2). 24 subjects had DPN. Foot ESC (μS) was significantly lower in the neuropathy group [53.5(25.1)] compared to No-DPN [77.0(7.9)] and HV [77.1(14.3), ANCOVA p<0.001, Fig 2a]. Similarly, hand ESC (μS) was also significantly lower in subjects with DPN [49.2(20.4)] compared to No-DPN [66.4(11.5)] and HV [64.4(14.3), ANCOVA p = 0.001, Fig 2b]. There was no significant difference in foot and hand ESC between No-DPN and HV groups (p = 0.72 and p = 0.46 respectively). Fig 3a displays the prognostic performance of SUDOSCAN ESC analyzed by Receiver Operating Curve (ROC) analysis when choosing the American Neurological Association diagnostic criteria for DPN. When choosing a foot ESC cut-off point of ≤ 77.0μS (optimal Youden index), sensitivity was 87.5%, specificity was 76.2% and the Youden index was 0.64. The area under the ROC curve was 0.85. The diagnostic performance of hand ESC was poorer in comparison to foot ESC (Table 3).

**Table 2:** Correlation of SUDOSCAN Measures with Vibration Detection Threshold, Nerve Conduction Studies and Cardiac Autonomic Function Tests.

	FESC	p	HESC	p	CAN-RS	p
Vibration JND	-0.49	0.001	-0.36	0.01		
Sural Amplitude (mV)	0.51	<0.001	0.43	0.003		
Peroneal Velocity (m/s)	0.75	<0.001	0.70	<0.001		
Peroneal Amplitude (mV)	0.55	<0.001	0.20	0.20		
Peroneal Distal Latency (mS)	0.32	0.03	0.01	0.92		
Tibial Distal Latency (mS)	-0.14	0.36	-0.14	0.35		
NCS Z-score	-0.62	<0.001	-0.52	<0.001		
R-R Deep Breathing	0.37	0.005	0.35	0.008	0.41	0.001
AFT Score	-0.41	0.001	-0.47	<0.001	-0.37	0.003

JND, Just Noticeable Difference; NCS, Nerve Conduction Studies; AFT, Autonomic Function Tests; FESC, Foot Electrochemical Skin Conductance; HESC, Hand Electrochemical Skin Conductance; CAN-RS, Cardiac Autonomic Neuropathy Risk Score.

**Table 3:** Receiver Operating Curve (ROC) analysis of classification of diabetic peripheral neuropathy (DPN) and cardiac autonomic neuropathy (CAN).

	FESC	HESC	CAN-RS
**DPN Classification**			
Sensitivity (%)	87.5	41.7	
Specificity (%)	76.2	100.0	
Youden Index	0.64	0.42	
Area Under ROC	0.85	0.74	
Cut-off point (μS)	77.0	42.0	
**CAN Classification**			
Sensitivity (%)	60.0	45.0	65.0
Specificity (%)	76.0	96.0	80.0
Youden Index	0.36	0.41	0.45
Area Under ROC	0.66	0.74	0.75
Cut-off point (μS for ESC, % for CAN-RS)	69.0	42.0	30.0

FESC, Foot Electrochemical Skin Conductance; HESC, Hand Electrochemical Skin Conductance; CAN-RS, Cardiac Autonomic Neuropathy Risk Score.

Next, we examined the differences in SUDOSCAN measures between subjects categorized according to CAN (n = 20) and No-CAN (n = 25, Table 1). Subjects with CAN had significantly lower foot [55.0(28.2), Fig 2c] and hand [53.5(19.6), Fig 2d] ESC compared to No-CAN [foot ESC, 72.1(12.2); hand ESC 64.9(14.4)] and HV groups (ANCOVA p<0.001 and 0.001 respectively). ROC analysis of CAN-RS to correctly classify T1DM subjects into CAN and No-CAN is shown in Fig 3b. The sensitivity was 65.0%, specificity was 80.0% and the Youden index was 0.45 with a CAN-RS cut-off point of ≥ 30.0% (optimal Youden index). The area under the ROC curve was 0.75. The diagnostic performance of hand and foot ESC was weaker in comparison (Table 3). DPN subjects [JND 20.0(4.1)] had higher vibration perception threshold compared to No-DPN [JND 15.7(3.3); p = 0.001]. We found a significant negative correlation between vibration JND with both FESC (ρ = -0.46, p = 0.002) and HESC (ρ = -0.40, p = 0.007). No adverse events or discomfort during and after measurement were reported.

## Conclusions

Currently, in our busy diabetic clinics we do not have a quantitative early marker of DPN. The measures we routinely use such as the 10g monofilament testing or peripheral neurological examination using other bedside instruments are crude, and detect the disease very late in its natural history. The situation is different for the early and accurate detection of retinopathy using the fundus camera as well as nephropathy by measuring microalbuminuria and eGFR. This has recently resulted in retinopathy no longer being the commenest casue of working age blindness in the UK. Unfortunately, by the time neuropathy is detected it is often very well established and consequently impossible to reverse and very difficult to halt the inexorable neuropathic process. Many of these patients end up in the foot clinic and have a very poor outcome with 5-year mortality close to 50% [27]. Early identifications of subjects with DPN using novel non-invasive methods will allow intensified treatment for blood glucose and cardiovascular risk factors in order to prevent or halt the progression of DPN. This is critically important as neuropathy is associated with much patient morbidity (foot ulceration, amputations, disabling pain etc.) and also mortality [2–4, 28]. Clearly, therefore the development of non-invasive, quick and sensitive measures of neuropathy has a most sound rationale.

In this study, that involved careful characterization of DPN using Gold-standard methodology according to recommendation of the American Academy of Neurology [18] unlike previous studies, we have demonstrated that SUDOSCAN has an excellent sensitivity (87.5%) and good specificity (76.2%) in detecting DPN. As expected foot ECS results detect neuropathy more sensitively than hand. Furthermore, foot and hand ESC demonstrated strong correlation with individual parameters and composite scores of nerve conduction. This demonstrates a close association between severity of SUDOSCAN measures with assessments of neuropathy severity. The area under the ROC curve showed a significant result for foot ECS (0.85, p<0.001). Our study has also showed that SUDOSCAN has a moderate sensitivity of 65.0% and specificity of 80.0% to correctly diagnose CAN. The area under the ROC curve was 0.75. These results are in accordance with study by Yajnik et al. [26] who used two abnormal Ewing tests as a reference for CAN, and found an area under the ROC curve for SUDOSCAN of 0.74, with a sensitivity of 92% and specificity of 49% [26]. Lower performance of CAN-RS in our study may be explained by the fact that this risk score was defined on previous studies performed in patients with T2DM [26].

A number of recent studies have looked at the potential utility of SUDOSCAN as a point-of-care device for detecting DPN. However, unlike the present study none of them used nerve conduction studies to characterize DPN. In a study involving 83 patients with both T1 and T2DM by Casellini et al. Sudoscan was found to have a similar sensitivity (78%) and specificity (92%) to our study in detecting neuropathy diagnosed by clinical exam (Neuropathy Impairment Score of the Lower Limbs—NIS-LL) with area under the curve in their ROC exactly the same as in our study at 0.85 [14]. Yajnik et al. [29] studied 265 diabetic patients and found that lower foot ESC was significantly associated both with increasing symptoms (MNSI A) and increasing score on physical abnormalities (MNSI B). Lower foot ESC was also significantly associated with increasing VPT (*P* < 0.01), and with a higher number of abnormal CAN results (*P* < 0.05). They concluded that sudomotor dysfunction testing may be a simple test to alert physicians to peripheral nerve and cardiac sympathetic dysfunction, highlighting that the ease of performance could make SUDOSCAN useful in the busy diabetic clinic [29]. Mayaudon et al. [15] measured sensitivity, specificity, and reproducibility of SUDOSCAN among 133 T2DM patients compared with 41 HVs. ESC showed a sensitivity of 75% and a specificity of 100%, with an area under the ROC curve of 0.88, similar to our present study. Another study involving 142 diabetes patients showed that reduction in foot ESC measurements correlated with an increasing VPT [16]. Bland–Altman plots indicated good reproducibility between two measurements [16]. Thus, our findings are in keeping with other recent studies. Even though the number of patients included in our study was small, unlike previous studies, all underwent careful characterization for peripheral neuropathy using Gold standard AAN criteria [18]. Furthermore, unlike previous studies we recruited T1DM subjects only to avoid a potential confound due to type of diabetes [30].

Currently, there are a number of validated methods of assessing sudomotor function [11]. However, none are suitable for use in the busy diabetic clinic due to the requirements of very specialized equipment, complicated patient preparation, highly trained technicians for test performance and/or interpretation, and prolonged testing time [14]. The potential use of SUDOSCAN appears to address all these disadvantages, as it is completely non invasive, can be performed in less than 5 minutes, doesn’t require any patient preparation or cooperation, and specialist training of the assessor is not necessary. The fact that it provides a ready objective and quantitative measure of DPN is particularly appealing, as this will allow the assessment of disease progression. In an international collaborative study we have recently shown that current clinical methods of detecting peripheral neuropathy are not reliable with significant variability even when performed by experts [10]. The use of such as quick, simple and objective measure of small-fibre neuropathy as screening measure in the busy diabetic clinic is therefore appealing. There are now a number of point of care devices that have been assessed for diagnosis and screening of DPN [31–35]. The concurrent validity results reported here for SUDOSCAN are comparable to these devices. The major limitation of many of these devices is the low specificity for detecting DPN. This suggests that although they may perform poorly on their own, in combination their performance may improve sufficiently to justify screening for DPN. Further studies will be required to assess this formally.

In conclusion, the results of our study suggest that peripheral sudomotor function; evaluated using SUDOSCAN is a reliable, objective and quantitative way which may be included as a screening tool for DPN. Prospective studies are required to investigate if abnormal SUDOSCAN results are predictive of the development of established DPN and hard outcomes such as foot ulceration.

## Abbreviations

AFT: Autonomic Function Tests
CAN: Cardiac Autonomic Neuropathy
CAN-RS: Cardiac Autonomic Neuropathy Risk Score
CART: Cardiovascular autonomic reflex tests
DPN: Diabetic peripheral neuropathy
FESC: Foot Electrochemical Skin Conductance
HV: Healthy volunteers
HESC: Hand Electrochemical Skin Conductance
JND: Just Noticeable Difference
NCS: Nerve Conduction Study
ROC: Receiver Operating Curve
T1DM: Type 1 diabetes
